# Genome Sequence of *Lactococcus lactis* subsp. *lactis* NCDO 2118, a GABA-Producing Strain

**DOI:** 10.1128/genomeA.00980-14

**Published:** 2014-10-02

**Authors:** Letícia C. Oliveira, Tessália D. L. Saraiva, Siomar C. Soares, Rommel T. J. Ramos, Pablo H. C. G. Sá, Adriana R. Carneiro, Fábio Miranda, Matheus Freire, Wendel Renan, Alberto F. O. Júnior, Anderson R. Santos, Anne C. Pinto, Bianca M. Souza, Camila P. Castro, Carlos A. A. Diniz, Clarissa S. Rocha, Diego C. B. Mariano, Edgar L. de Aguiar, Edson L. Folador, Eudes G. V. Barbosa, Flavia F. Aburjaile, Lucas A. Gonçalves, Luís C. Guimarães, Marcela Azevedo, Pamela C. M. Agresti, Renata F. Silva, Sandeep Tiwari, Sintia S. Almeida, Syed S. Hassan, Vanessa B. Pereira, Vinicius A. C. Abreu, Ulisses P. Pereira, Fernanda A. Dorella, Alex F. Carvalho, Felipe L. Pereira, Carlos A. G. Leal, Henrique C. P. Figueiredo, Artur Silva, Anderson Miyoshi, Vasco Azevedo

**Affiliations:** a Laboratory of Cellular and Molecular Genetics, Institute of Biologic Sciences, Federal University of Minas Gerais, Belo Horizonte, MG, Brazil; b Institute of Biologic Sciences, Federal University of Pará, Belém, PA, Brazil; c AQUACEN, National Reference Laboratory for Aquatic Animal Diseases, Ministry of Fisheries and Aquaculture, Federal University of Minas Gerais, Belo Horizonte, MG, Brazil

## Abstract

*Lactococcus lactis* subsp. *lactis* NCDO 2118 is a nondairy lactic acid bacterium, a xylose fermenter, and a gamma-aminobutyric acid (GABA) producer isolated from frozen peas. Here, we report the complete genome sequence of *L. lactis* NCDO 2118, a strain with probiotic potential activity.

## GENOME ANNOUNCEMENT

Lactic acid bacteria (LAB), in general, acquire energy from the conversion of sugars into lactic acid (1) and are used for production of many fermented products, such as cheese, yogurt, butter, and wine. Food conservation is due to the medium acidification and production of molecules that inhibit the growth of undesirable microbiota, contributing to the development of desirable organoleptic properties in the final product (2). Moreover, some specific LAB strains produce bioactive molecules such as gamma-aminobutyric acid (GABA) (3), a product of glutamate decarboxylation by the glutamic acid decarboxylase (GAD) enzyme. Usually, GABA acts by modulating the central nervous system, contributing to smooth muscle relaxation and presenting hypotensor activity (4). Also, GABA can immunomodulate the immune system (5). Therefore, GABA-producing bacteria generally present probiotic properties (6). *Lactococcus lactis* NCDO 2118 is a nondairy strain, a xylose fermenter (a common trait of plant-associated strains), and a GABA producer isolated from frozen peas (6, 7).

*L. lactis* NCDO 2118 was sequenced three times, due to assembling complexity. First, the genome was decoded with the SOLiD 5500 platform with mate-paired libraries, generating a total of 5,133,057,360 bp, (coverage of 2,053 times). The reads were subjected to a Phred 20 quality filter using Quality Assessment software (8) and assembled with the CLC Genomics Workbench, generating a total of 1,641 overlapping sequences. These sequences were removed with the Simplifier (9), ordered and oriented based on the reference *L. lactis* KF147 genome sequence (a plant-associated strain, accession number CP001834). Then manual curation was performed using Artemis (10), and SSPACE (11) and Gapfiller (12) were used to generate the scaffold and resolve gaps, respectively. At the end of curation and sequence assembly, a total of 409 scaffolds (2,874,854 bp) were obtained.

*L. lactis* NCDO 2118 was then decoded with the Ion PGM platform with fragment libraries generating a total of 187,303,001 bp (coverage of ~71 times). Genome assembly was performed using Mira 3.9 (13), and the assembled genome sequence was reference aligned with CONTIGuator (14). The redundant overlapping sequences were removed with “in-house scripts,” closing the remnant gaps. Annotation and frameshifts curation were then performed using Artemis and CLC, reducing the initial 1821 pseudogenes to 480.

Finally, the DNA was sequenced using the Ion Torrent PGM with fragment libraries, yielding a total of ~1,249,154,478 bp (coverage of 474 times). Assembly was performed with Mira 4.0.1 and Newbler 2.9 (15). We used CONTIGuator and FGAP 1.7 (16) to perform the alignment and gap closure steps, respectively. We followed the same previously explained steps for annotation and frameshift curation, reducing the pseudogenes to 52.

The complete genome of *L. lactis* NCDO 2118 consists of a single circular chromosome of 2,554,693 bp, containing 2,386 coding sequences (CDS), which had 52 pseudogenes, 66 tRNA genes, and 6 rRNA operons, with a G+C content of 34.9%. There is one plasmid, pNCDO2118 (37,571 bp), with 48 CDS, from which 4 are pseudogenes with a G+C content of 32.33%.

### Nucleotide sequence accession numbers.

The *Lactococcus lactis* NCDO 2118 chromosome and the plasmid were deposited at DDBJ/EMBL/GenBank under the accession numbers CP009054 and CP009055, respectively.
